# Phase-Controlled Synthesis of Alloyed (CdS)_x_(CuInS_2_)_1−x_ Nanocrystals with Tunable Band Gap

**DOI:** 10.3390/nano15211661

**Published:** 2025-11-01

**Authors:** Bingqian Zu, Song Chen, Liping Bao, Yingjie Liu, Liang Wu

**Affiliations:** Key Laboratory of Functional Molecular Solids, Ministry of Education, School of Chemistry and Materials Science, Anhui Normal University, Wuhu 241000, China

**Keywords:** alloyed (CdS)_x_(CuInS_2_)_1−x_ nanocrystals, wurtzite, zinc-blende, composition, band gap

## Abstract

Phase and band gap engineering of (CdS)_x_(CuInS_2_)_1−x_ nanomaterials is critical for their potential applications in photovoltaics and photocatalysis, yet it remains a challenge. Here, we report a precursor-mediated colloidal method for phase-control synthesis of alloyed (CdS)_x_(CuInS_2_)_1−x_ nanocrystals with tunable band gap. When CuCl, InCl_3_, and Cd(AC)_2_·2H_2_O are used as the respective cation sources, wurtzite-structured alloyed (CdS)_x_(CuInS_2_)_1−x_ nanocrystals can be synthesized with a tunable optical band gap ranging from 1.56 to 2.45 eV by directly controlling the molar ratio of the Cd precursor. Moreover, using Cu(S_2_CNEt_2_)_2_, In(S_2_CNEt_2_)_3_, and Cd(S_2_CNEt_2_)_2_ as cation sources results in alloyed (CdS)_x_(CuInS_2_)_1−x_ nanocrystals with a zinc-blende structure, demonstrating that the optical band gap of these nanocrystals can be compositionally tuned from 1.50 to 1.84 eV through precisely adjusting the molar ratio of Cd precursor. The results were validated through a comprehensive characterization approach employing XRD, TEM, HRTEM, STEM-EDS, XPS, UV-vis-NIR absorption spectroscopy, and Mott–Schottky analysis.

## 1. Introduction

Colloidal semiconductor nanocrystals with tunable band gaps have gained significant attention as functional materials for a wide range of applications, including light-emitting diodes, solar cells, and photocatalysis [1,2,3,4]. Generally, the band gap of colloidal semiconductor nanocrystals can be regulated by changing the nanocrystal size due to the quantum size effect or by modifying the composition in alloyed nanocrystals [5]. However, adjusting the band gap solely by varying particle size limits the extension of absorption range. Moreover, size-dependent properties tend to diminish when nanocrystals are deposited onto thin films and undergo thermal treatment for practical applications [6].

In contrast, controlling the composition represents a more feasible way for fabricating semiconductor thin films with adjustable band gaps [7,8,9]. By varying the composition of multicomponent nanocrystals, it becomes straightforward to achieve a range of band gaps, even when the size remains constant. DFT calculation indicates that synthesized alloyed materials are an effective approach for tuning band gaps [10,11,12,13]. Based on these, a great variety of alloyed semiconductor nanocrystals with a tunable composition and band gap have been extensively investigated, such as Zn_x_Cd_1−x_S, CuInS_x_Se_1−x_, CuInS_2_-ZnS, CuIn_x_Ga_1−x_S_2_, and AgInS_2_-ZnS [14,15,16,17,18,19].

Recently, CuInS_2_ and its alloyed nanocrystals with II-VI semiconductors have been extensively studied due to their properties as direct band gap semiconductors with high absorption coefficients [20,21,22]. Alloying CuInS_2_ (Eg = 1.5 eV) with CdS (Eg = 2.5 eV) enables the formation of a series of nanocrystals with adjustable band gaps by controlling the ratio of CdS to CuInS_2_ during synthesis [23,24]. The tunability is crucial for applications in optoelectronics, such as solar cells and photodetectors, where specific light absorption and emission spectra are required [25,26]. However, synthesizing homogeneous (CdS)_x_(CuInS_2_)_1−x_ alloys with arbitrary compositions remains challenging due to their inherent phase disparity.

In addition, crystal structure has been widely recognized as a dominant characteristic of inorganic nanocrystals, offering effective approaches to modulate their fundamental properties such as optical, electronic, and mechanical characteristics [27,28,29]. This modulation enables the precise tailoring of nanocrystals’ behavior, which is crucial for various technological advancements. More recently, CuInS_2_ nanocrystals with a chalcopyrite (CP) phase and a metastable wurtzite (WZ) structure have been successfully synthesized [30,31,32]. This phase diversity of CdS and CuInS_2_ offers the potential for synthesizing alloyed (CdS)_x_(CuInS_2_)_1−x_ nanocrystals with a tunable crystal structure, which is crucial for achieving optimal electronic and optical performance.

Therefore, the manipulation of phase and band gap properties in alloyed (CdS)_x_(CuInS_2_)_1−x_ nanocrystals offers significant potential for advanced optoelectronic and photocatalytic applications. Herein, we develop a precursor-mediated colloidal method for the phase-controlled synthesis of alloyed (CdS)_x_(CuInS_2_)_1−x_ nanocrystals with tunable bandgaps. Using CuCl, InCl_3_, and Cd(AC)_2_·2H_2_O as cation precursors results in the formation of (CdS)_x_(CuInS_2_)_1−x_ nanocrystals with a WZ structure, whereas employing Cu(S_2_CNEt_2_)_2_, In(S_2_CNEt_2_)_3_, and Cd(S_2_CNEt_2_)_2_ as cation sources enables the synthesis of zinc-blende (ZB) (CdS)_x_(CuInS_2_)_1−x_ nanocrystals. Their microstructural characteristics were meticulously examined utilizing transmission electron microscopy (TEM) and high-resolution TEM (HRTEM), complemented by X-ray diffraction (XRD) analysis. Furthermore, the electronic band structures were systematically estimated through a synergistic approach involving UV−visible−near-infrared (UV−vis−NIR) absorption spectroscopy and Mott−Schottky analysis. As a result, increasing the Cd precursor ratio enables the synthesis of alloyed nanocrystals with band gaps ranging from 1.56 to 2.45 eV for the WZ phase and from 1.50 to 1.84 eV for the ZB phase.

## 2. Materials and Methods

### 2.1. Chemicals

Hexane (97%), Ethanol absolute (99.7%), Copper (I) chloride (CuCl, 97.0%), Cadmium acetate dihydrate (Cd(AC)_2_·2H_2_O, 98.0%) and 1-Dodecanethiol (1-DDT, 98.0%) were purchased from Sinopharm Chemical Reagent Co. Ltd. (Shanghai, China). Indium Chloride (InCl_3_, 98%), Copper nitrate trihydrate (Cu(NO_3_)_2_·3H_2_O, 99%), Cadmium nitrate tetrahydrate (Cd(NO_3_)_2_·4H_2_O, 99%), and Indium(III) nitrate tetrahydrate (In(NO_3_)_3_·4H_2_O, 99.9%) were purchased from Macklin reagent Inc. (Shanghai, China). Sodium diethyldithiocarbamate trihydrate (NaS_2_CNEt_2_, 99.0%), Sodium sulfate anhydrous (Na_2_SO_4_, 99%), Oleylamine (OLA, 80–90%), and 1-Octadecene (ODE, 99.0%) were purchased from Aladdin Reagent Co. Ltd. (Shanghai, China). All chemical reagents were used as received without further purification.

### 2.2. Synthesis of WZ Alloyed (CdS)_x_(CuInS_2_)_1−x_ Nanocrystals

CuCl (0.5 mmol), InCl_3_ (0.5 mmol), Cd(AC)_2_·2H_2_O (1.5 mmol), OLA (10 mL), and 1-DDT (1.0 mL) were sequentially added to a 50 mL three-neck flask. The mixture was heated to 100 °C with a heating rate of 10 °C/min and degassed at this temperature for 10 min. Subsequently, the reaction temperature was increased to 240 °C at the same heating rate and held for 1 h under ambient atmospheric conditions. After the completion of the reaction, the three-necked flask was allowed to cool naturally. The alloyed nanocrystals were obtained by centrifugation. The obtained nanocrystals were washed twice with hexane and ethanol and subsequently dispersed in hexane. WZ alloyed (CdS)_x_(CuInS_2_)_1−x_ nanocrystals with varying compositions were synthesized using different amounts Cd(AC)_2_·2H_2_O under identical reaction conditions (Table S1).

### 2.3. Synthesis of ZB Alloyed (CdS)_x_(CuInS_2_)_1−x_ Nanocrystals

Cu(S_2_CNEt_2_)_2_ (0.1 mmol), In(S_2_CNEt_2_)_3_ (0.1 mmol), and Cd(S_2_CNEt_2_)_2_ (0.3 mmol) were dissolved in 4 mL of OLA, 4 mL of DDT, and 3 mL of ODE in a three-neck flask at room temperature. The temperature of the reaction solution was subsequently increased to 80 °C over a period of 6 min, followed by degassing at this temperature for 10 min. After that, the reaction temperature was raised to 250 °C with a heating rate of 10 °C/min and maintained for 1 h under ambient atmospheric conditions. Lastly, the ZB alloyed nanocrystals were collected and purified in the same way as WZ alloyed nanocrystals. ZB alloyed (CdS)_x_(CuInS_2_)_1−x_ nanocrystals with varying compositions were synthesized using different amounts of Cd(S_2_CNEt_2_)_2_ under identical reaction conditions (Table S2).

### 2.4. Materials Characterizations

The crystalline structure of the products was characterized by X-ray powder diffractometer (PXRD, SmartLab 9 kw) equipped with graphite-monochromated Cu KR radiation (λ = 1.54056 Å). Nanocrystals dispersed in hexane were dropped onto gold-mesh carbon support membrane and HRTEM (Tecnai G220 FEI) was used to acquire TEM and HRTEM images, along with scanning transmission electron microscopy–energy dispersive X-ray spectroscopy (STEM-EDS) element mapping analyses. UV-Vis-NIR photometers (UV-3600iPlus, Shimadzu) were utilized to measure the spectra of nanocrystals dispersed in chloroform at room temperature. The chemical states of the obtained nanocrystals were systematically characterized using X-ray photoelectron spectroscopy testing (XPS, Thermo Scientific K-Alpha). The Mott–Schottky curves were conducted using a CHI 760E electrochemical workstation in a single-chamber electrolytic cell. The experimental setup consists of a (CdS)_x_(CuInS_2_)_1−x_ working electrode, a Pt foil counter electrode, and a Ag/AgCl reference electrode, all immersed in a 0.5 M Na_2_SO_4_ electrolyte solution.

## 3. Results and Discussion

**Figure 1:**
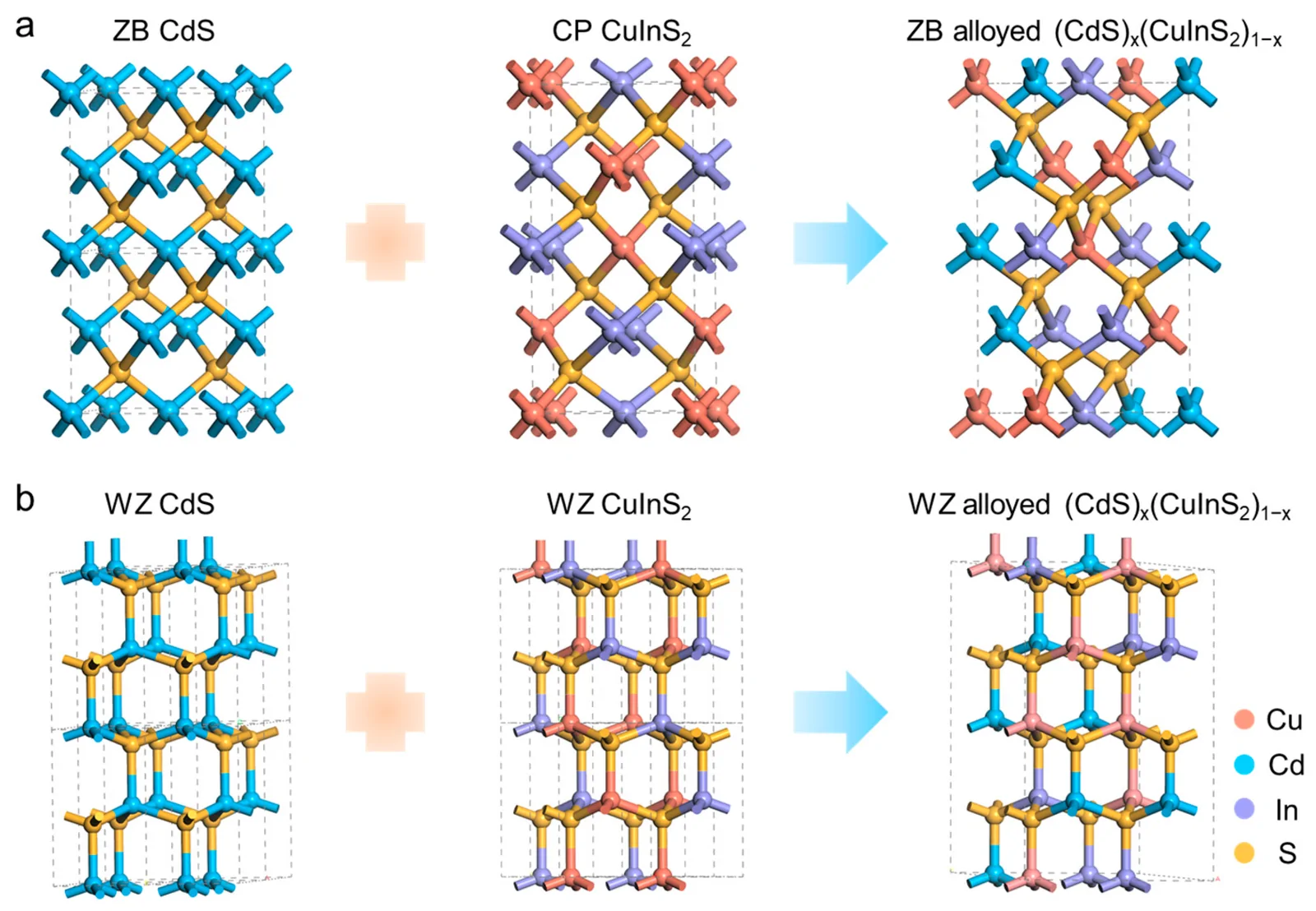
(**a**) Double unit cells of ZB CdS, CP CuInS_2_, and ZB alloyed (CdS)_x_(CuInS_2_)_1−x_. (**b**) Double unit cells of WZ CdS, WZ CuInS_2_, and WZ alloyed (CdS)_x_(CuInS_2_)_1−x_.

Ternary CuInS_2_ can be regarded as being structurally derived from binary CdS [33]. There are three primary structural pathways from binary CdS to ternary CuInS_2_. Among them, the CP phase represents the thermodynamically stable structure, whereas the ZB and WZ structures are classified as metastable. Furthermore, the CP phase can be viewed as a cation-ordered variant of the ZB structure. CdS shares the same crystal structure as ZnS, and the alloyed ZnS-CuInS_2_ nanocrystals with WZ and ZB structures have been successfully synthesized across a range of compositions [34,35,36]. Therefore, CP CuInS_2_ can be alloyed with ZB CdS to produce ZB (CdS)_x_(CuInS_2_)_1−x_ nanocrystals (Figure 1a). Similarly, WZ CuInS_2_ can be alloyed with WZ CdS to yield WZ (CdS)_x_(CuInS_2_)_1−x_ nanocrystals (Figure 1b).

Meanwhile, phase-controlled synthesis of alloyed (CdS)_x_(CuInS_2_)_1−x_ nanocrystals with tunable band gap involves meticulously regulating the reaction conditions to achieve precise control over the crystalline phases and composition ratios. Herein, a precursor-mediated colloidal approach was used to synthesize alloyed (CdS)_x_(CuInS_2_)_1−x_ nanocrystals (Figure S1). For the synthesis of WZ alloyed (CdS)_x_(CuInS_2_)_1−x_ nanocrystals, CuCl, InCl_3_, Cd(AC)_2_·2H_2_O, together with OLA and 1-DDT, were utilized as precursors and capping agents. In contrast, ZB alloyed (CdS)_x_(CuInS_2_)_1−x_ nanocrystals were synthesized using Cu(S_2_CNEt_2_)_2_, In(S_2_CNEt_2_)_3_, and Cd(S_2_CNEt_2_)_2_ as precursors, with OLA, 1-DDT, and ODE serving as solvents and ligands.

**Figure 2:**
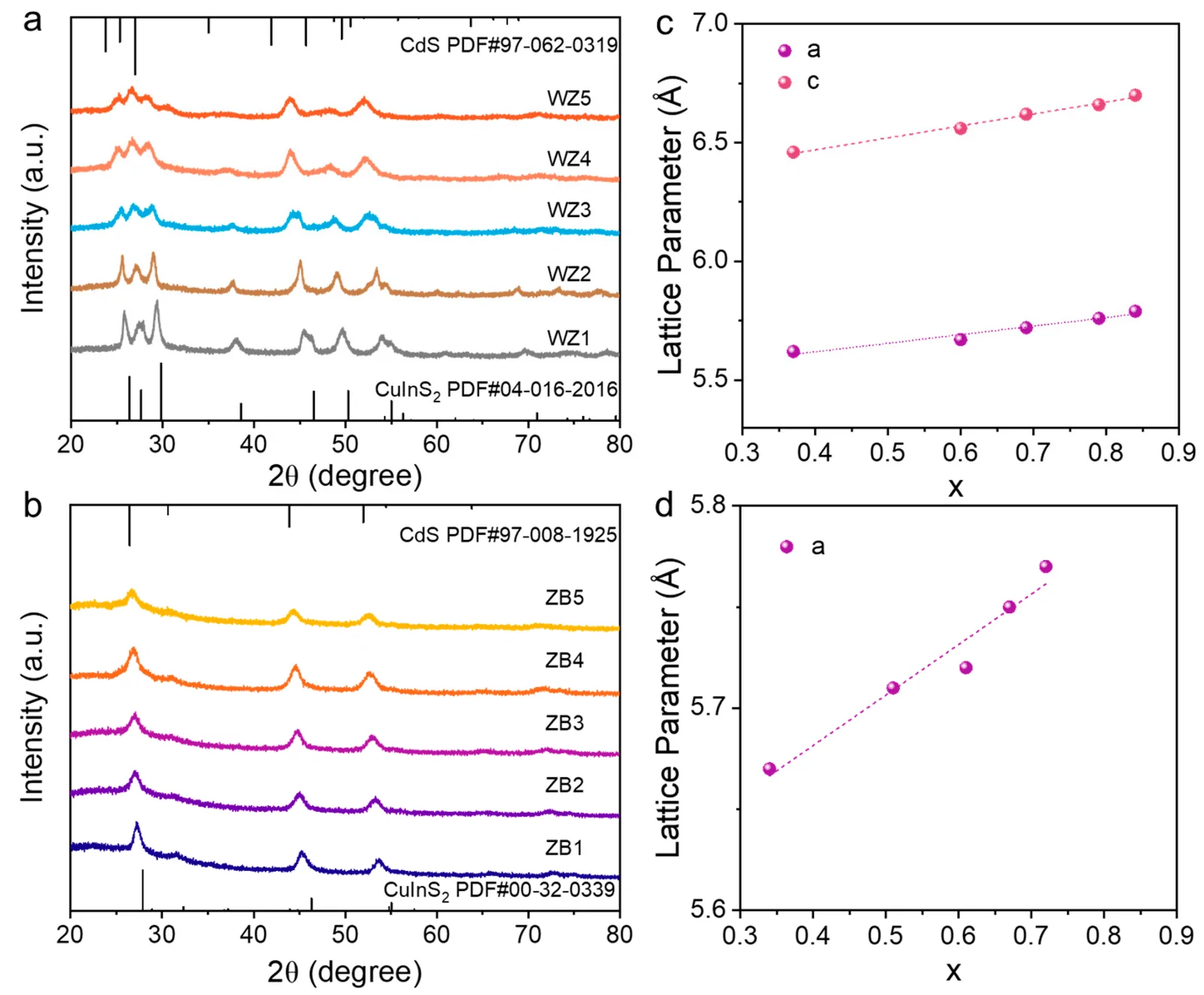
(**a**,**b**) XRD patterns of the synthesized WZ and ZB alloyed (CdS)_x_(CuInS_2_)_1−x_ nanocrystals with various compositions. (**c**) The lattice parameters a and c of the WZ alloyed (CdS)_x_(CuInS_2_)_1−x_ nanocrystals with various compositions. (**d**) The lattice parameter a of the ZB alloyed (CdS)_x_(CuInS_2_)_1−x_ nanocrystals with various compositions. For comparison purposes, XRD patterns of the WZ and ZB CdS (top patterns) and WZ and CP CuInS_2_ (bottom patterns) are also provided.

The crystal phase of the alloyed (CdS)_x_(CuInS_2_)_1−x_ nanocrystals was analyzed using XRD. Figure 2a displays the XRD patterns of WZ alloyed (CdS)_x_(CuInS_2_)_1−x_ nanocrystals with various compositions (Table S1), along with the standard diffraction peaks of WZ CdS and WZ CuInS_2_. All observed XRD patterns of the synthesized (CdS)_x_(CuInS_2_)_1−x_ nanocrystals align with the hexagonal wurtzite crystal structure. Figure 2b illustrates the XRD patterns of the synthesized ZB alloyed (CdS)_x_(CuInS_2_)_1−x_ nanocrystals with differing compositions (Table S2), confirming that all observed XRD patterns match the cubic zinc-blende crystal structure. For both the WZ and ZB alloyed (CdS)_x_(CuInS_2_)_1−x_ nanocrystals, the diffraction peaks exhibit a gradual shift toward lower diffraction angles as the ratio of Cd precursor increases, and the corresponding lattice parameters increase accordingly (Figure 2c,d, Tables S3 and S4), suggesting the successful formation of (CdS)_x_(CuInS_2_)_1−x_ solid solutions, rather than a mere physical mixture of CdS and CuInS_2_ [37]. The peak shift is consistent with the difference in ionic radius, as Cd^2+^ (0.97 Å) has a lager ionic radius than In^3+^ (0.76 Å) and Cu^+^ (0.74 Å) [38].

**Figure 3:**
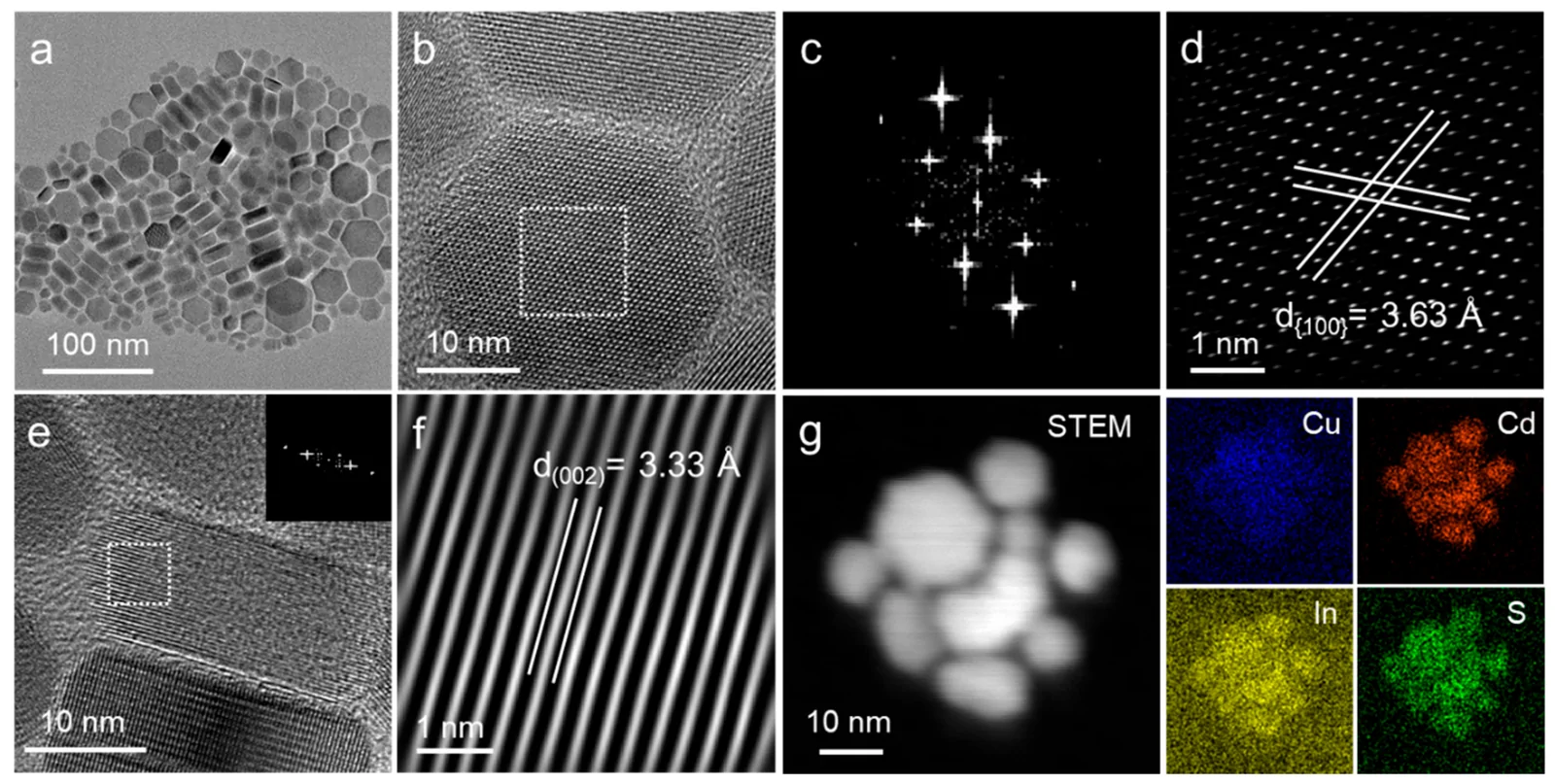
Characterization of WZ3 nanocrystals. (**a**) TEM image. (**b**,**e**) HRTEM images of a nanocrystal oriented perpendicularly and horizontally on gold grid, respectively. The insert shows the FFT pattern from the selected area in (**e**). (**c**) The corresponding FFT pattern from the selected area in (**b**). (**d**,**f**) The inversed FFT images from (**c**) and the insert in (**e**), respectively. (**g**) STEM image and EDS-elemental mapping.

Figure 3a and Figure S2 show the TEM images of the synthesized WZ alloyed (CdS)_x_(CuInS_2_)_1−x_ nanocrystals with varying composition. All the obtained WZ alloyed nanocrystals exhibit a hexagonal plate-like morphology. The dimensions of samples WZ1–5 are within the ranges 12.31 ± 0.78, 15.36 ± 0.38, 14.79 ± 0.54, 18.31 ± 0.29, and 10.30 ± 0.22 nm, respectively (Figure S3). Figure 3b,e display the HRTEM images of a WZ3 nanocrystal oriented perpendicularly and horizontally on gold grids, respectively, confirming its single-crystalline nature. The corresponding fast Fourier transform (FFT) pattern (Figure 3c) obtained from the selected area in b further confirms the wurtzite crystal structure of the WZ3 nanocrystal. The measured *d*-spacing of 3.63 Å corresponds to the (100) plane of the WZ (CdS)_x_(CuInS_2_)_1−x_ (Figure 3d). Additionally, a lattice fringe is 3.33 Å is observed in Figure 3f, which is consistent with the (002) planes of WZ (CdS)_x_(CuInS_2_)_1−x_. To further analyze the compositional homogeneity, STEM-EDS was performed on the obtained WZ3 NCs. As shown in Figure 3g, the elements Cu, Cd, In, and S are uniformly distributed across randomly selected WZ3 nanocrystals, with no significant elemental segregation or clustering detected. Moreover, we investigated the influence of type of Cd source the morphology and structure of the WZ alloyed (CdS)_x_(CuInS_2_)_1−x_ nanocrystals. Taking the synthesis of WZ1 nanocrystals as an example, when the Cd(AC)_2_·2H_2_O is replaced by Cd(acac)_2_ and CdCl_2_, the resulting WZ1 nanocrystals, although maintaining the WZ structure (Figure S4), exhibit a morphology consisting of a mixture of nanoplates and nanoparticles (Figure S5).

**Figure 4:**
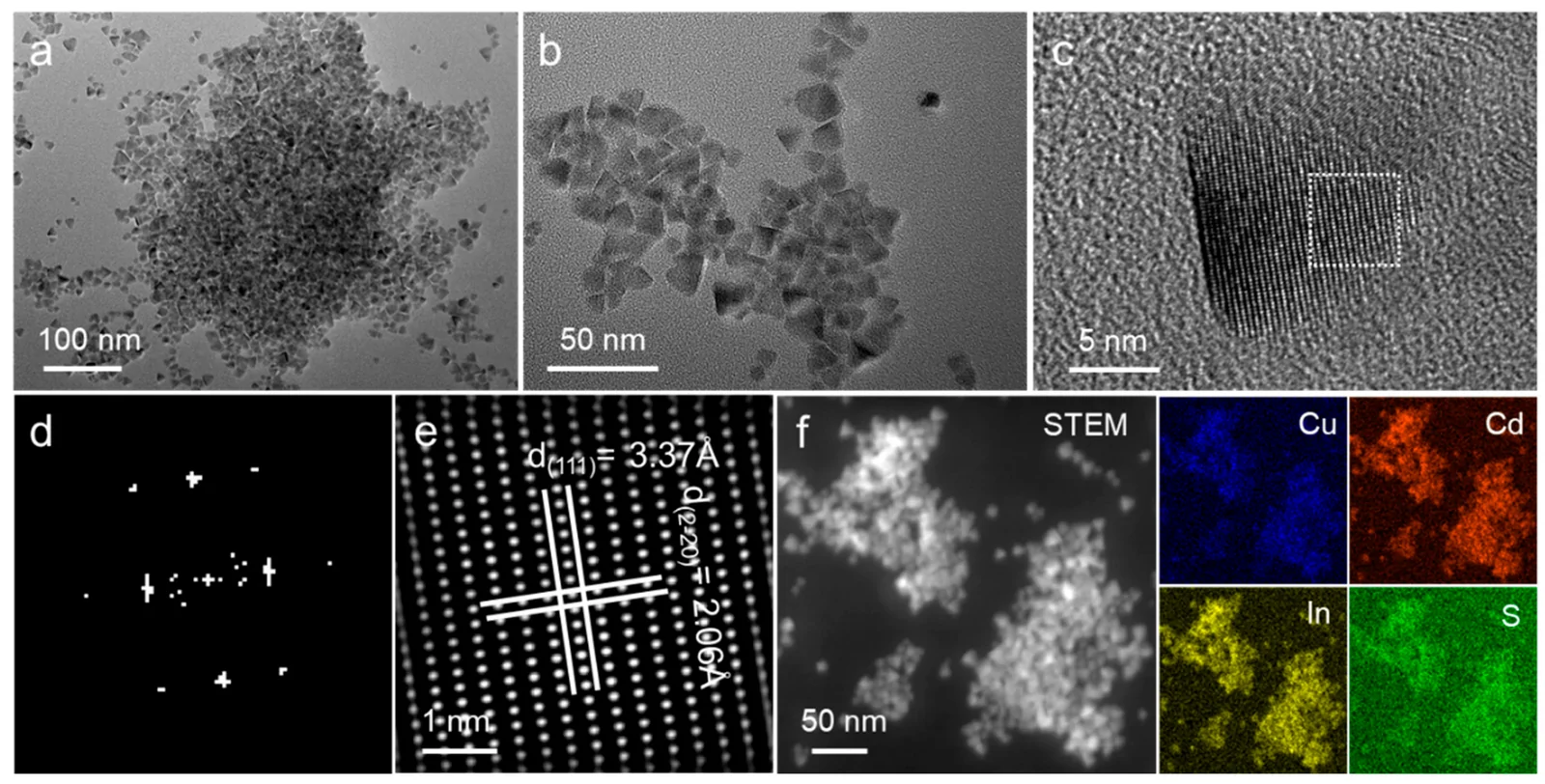
Characterization of ZB3 nanocrystals. (**a**,**b**) TEM images. (**c**) HRTEM images. (**d**) The FFT pattern from the selected area (dashed box) in (**c**). (**e**) The inversed FFT image from (**d**). (**f**) STEM image and EDS-elemental mapping.

Figure 4a,b and Figure S6 presents the typical TEM images of the synthesized ZB (CdS)_x_(CuInS_2_)_1−x_ nanocrystals with varying composition. All obtained ZB alloyed nanocrystals exhibit a pyramidal morphology. The dimensions of samples ZB1–5 are within the ranges 11.34 ± 0.37, 12.44 ± 0.23, 10.93 ± 0.30, 11.66 ± 0.27, and 10.69 ± 0.38 nm, respectively (Figure S7). Figure 4c,d display the HRTEM image and the corresponding FFT pattern of a ZB3 nanocrystal, respectively, verifying the cubic crystal nature of ZB3 nanocrystal. Clear lattice fringes are observed in Figure 4c, and two lattice planes of the zinc-blende crystal structure, namely (111) and (2–20), are identified (Figure 4e). STEM-EDS element mapping (Figure 4f) demonstrates a uniform distribution of the elements Cu, Cd, In, and S across randomly selected ZB3 nanocrystals, with no noticeable elemental segregation or clustering observed. Additionally, we examined the influence of the ratio of OLA and 1-DDT, as well as the solvent volume, on the synthesis of ZB alloyed (CdS)_x_(CuInS_2_)_1−x_ nanocrystals. Taking the synthesis of ZB nanocrystals as case study, when 10 mL of OLA and 1 mL of 1-DDT are employed as solvent, the resulting ZB4 nanocrystals exhibit negligible morphological changes despite a decrease in crystallinity (Figures S8a–c and S9a). However, increasing the solvent volume led to an increase in the size of the ZB4 nanocrystals, accompanied by a broader size distribution (Figures S8d–f and S9b).

**Figure 5:**
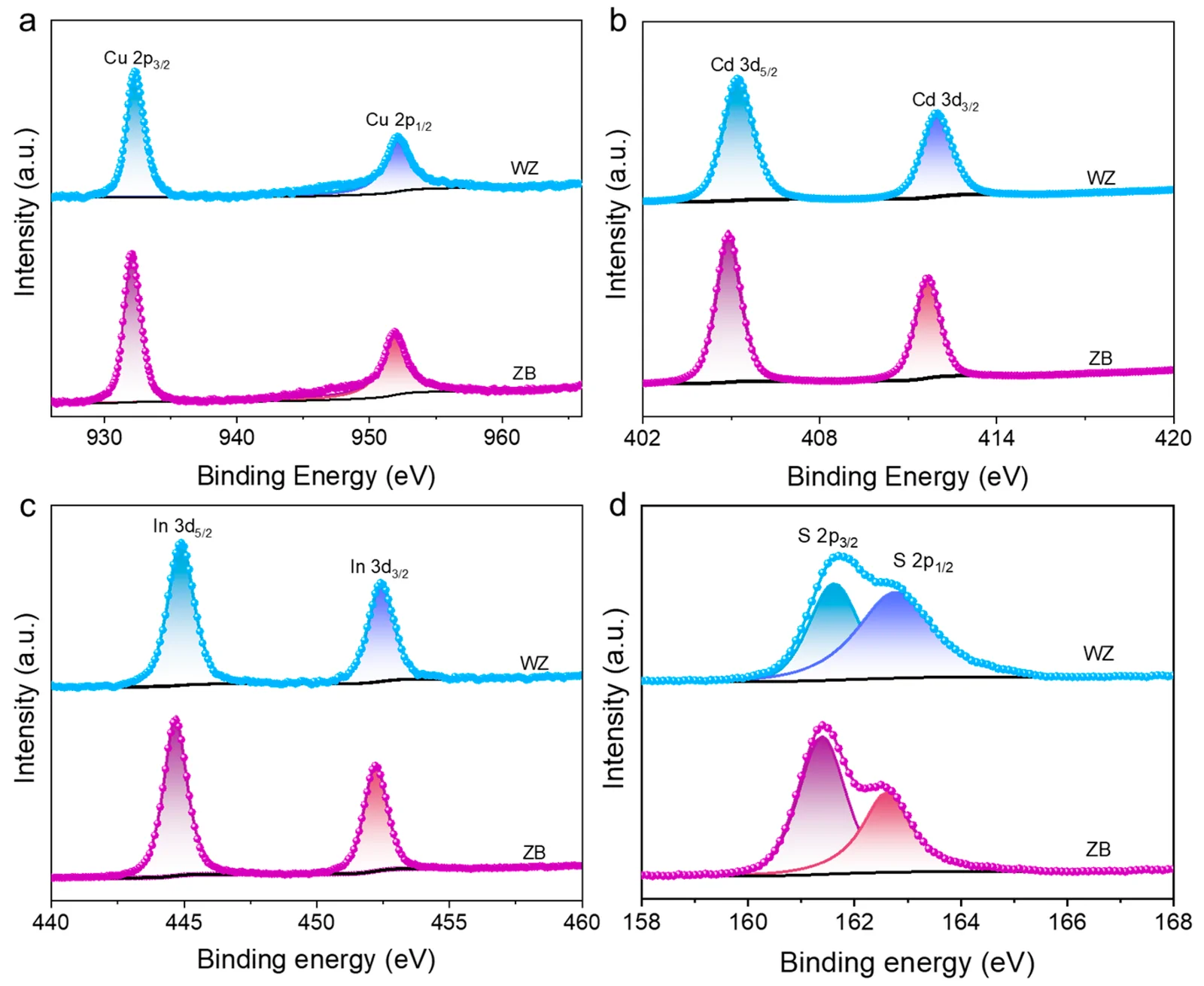
XPS spectra of WZ3 and ZB3 nanocrystals. (**a**) Cu 2p. (**b**) Cd 3d. (**c**) In 3d. (**d**) S 2p.

XPS was further used to investigate the surface electron state of the synthesized alloyed (CdS)_x_(CuInS_2_)_1−x_ nanocrystals. As illustrated in Figure S10a, the detection results confirm the presence of Cu, Cd, In, and S elements in both WZ3 and ZB3 nanocrystals. The binding energy of C 1s peak at 284.8 eV is used for the reference for charge correction (Figure S10b). Figure 5 presents the high-resolution XPS spectra of Cu 2p, Cd 3d, In 3d, and S 2p for WZ3 and ZB3 nanocrystals. For WZ3 nanocrystals, the Cu 2p spectrum (Figure 5a) reveals the Cu 2p_3/2_ and Cu 2p_1/2_ peaks at binding energies of 932.2 and 952.0 eV, respectively, with a separation of 19.8 eV, which is consistent with Cu(I) [39]. The Cd 3d spectrum (Figure 5b) exhibits two peaks at 405.2 eV and 411.9 eV, with a splitting of 6.7 eV, indicating the Cd(II) oxidation state [40]. Similarly, the In 3d peaks are observed at 444.9 eV and 452.4 eV, showing a separation of 7.5 eV, which confirms the presence of In(III) (Figure 5c) [41]. The S 2p spectrum (Figure 5d) displays a spin–orbit doublet fitted into two peaks with an energy difference of 1.0 eV, corresponding to sulfide species [38]. The elements in ZB alloyed nanocrystals exhibit the same oxidation states as those in WZ alloyed nanocrystals. The minor differences between the XPS spectra of WZ3 and ZB3 may be attributed to variations in their crystal structures.

**Figure 6:**
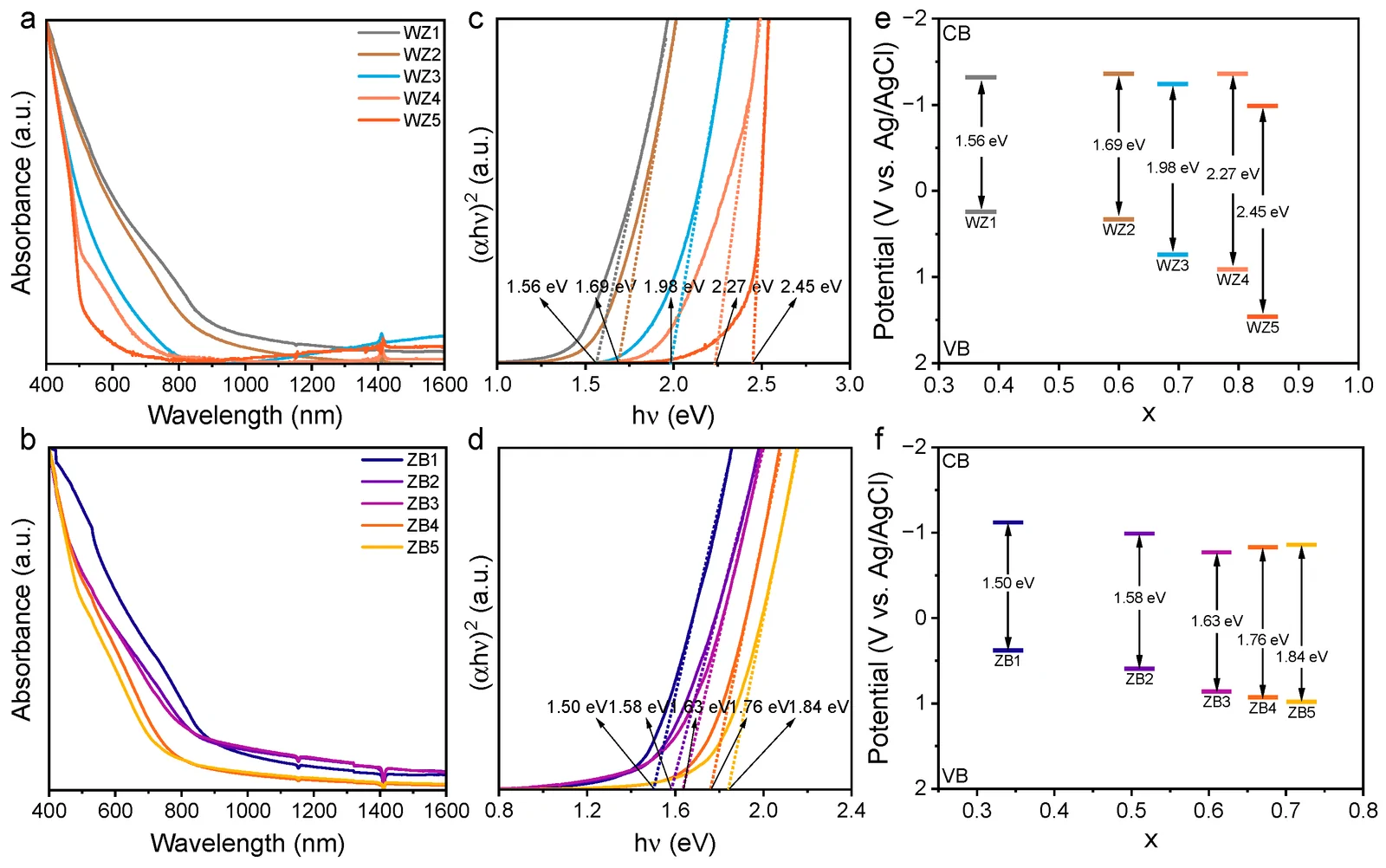
(**a**,**b**) UV-vis-NIR absorption spectra of WZ and ZB alloyed (CdS)_x_(CuInS_2_)_1−x_ nanocrystals with varying compositions, respectively. (**c**,**d**) Plots of linear extrapolation of (*αhν*)^2^ versus photon energy (*hν*) of WZ and ZB alloyed (CdS)_x_(CuInS_2_)_1−x_ nanocrystals with varying compositions, respectively. (**e**,**f**) The schematic diagrams of the electronic band positions of WZ and ZB alloyed (CdS)_x_(CuInS_2_)_1−x_ nanocrystals with varying compositions, respectively.

UV-Vis-NIR absorption spectroscopy was used to characterize the optical properties of the synthesized WZ and ZB alloyed (CdS)_x_(CuInS_2_)_1−x_ nanocrystals. As shown in Figure 6a,b, both the WZ and ZB alloy nanocrystals exhibit a broad and tunable absorption range. The band-edge absorption of the alloyed nanocrystals shows a gradual blue shift toward shorter wavelength with an increasing ratio of Cd precursor, which can be attributed to the wider band gap of CdS compared to that of CuInS_2_. Figure S11 presents photographs of the WZ (left) and ZB (right) alloyed (CdS)_x_(CuInS_2_)_1−x_ nanocrystals with systematically increasing ratio of Cd precursor. A progressive color transition from black to yellow is observed, indicating an increase in the band gaps. The optical band gaps of these alloyed nanocrystals were determined by plotting (*αhν*)^2^ vs. *hν*, extrapolating the linear portion to the x axis [39,42,43]. This analysis reveals that increasing the ratio of Cd precursor in the WZ alloyed (CdS)_x_(CuInS_2_)_1−x_ nanocrystals lead to a consistent increase in the band gap energies from 1.56 to 2.45 eV (Figure 6c), and in ZB alloyed (CdS)_x_(CuInS_2_)_1−x_ nanocrystals, the band gap energies increase consistently from 1.50 to 1.84 eV (Figure 6d).

It is essential to thoroughly understand the band energy structure of a material prior to utilizing its potential applications in photocatalysis and photovoltaic cells [44,45,46]. The alloying of CdS and CuInS_2_ is expected to modify the orbital characteristics of the valence band (VB) and conduction band (CB), thereby shifting their energy levels relative to carrier transport processes, which can influence the overall performance in practical applications [47,48]. Alternating-current (AC) impedance measurements employed in Mott−Schottky (M-S) analysis serve as a powerful and insightful technique for effectively addressing this issue [28,49]. The AC impedance measurements were conducted on each WZ and ZB alloyed (CdS)_x_(CuInS_2_)_1−x_ electrode for M−S analysis to determine the flat-band potentials (E_fb_) experimentally. As shown in Figures S12 and S13, the M-S plots of all alloyed (CdS)_x_(CuInS_2_)_1−x_ electrodes in both WZ and ZB structures display negative slopes, confirming their p-type characteristics [50,51]. The E_fb_ was employed to estimate the position of the VB maximum. Given the proximity of the Fermi level to the majority carrier edge, it is conventional to assign E_fb_ to the valence band position in the case of p-type materials. The schematic diagrams presented in Figure 6e,f illustrate the electronic band positions of WZ and ZB alloyed (CdS)_x_(CuInS_2_)_1−x_ nanocrystals, revealing a variation and alignment pattern consistent with that previously reported for alloyed (ZnS)_x_(CuInS_2_)_1−x_ nanocrystals.

## 4. Conclusions

In summary, we have successfully developed a precursor-mediated colloidal synthesis approach that allows precise control over both the phase and composition of alloyed (CdS)_x_(CuInS_2_)_1−x_ nanocrystals. Using CuCl, InCl_3_, and Cd(AC)_2_·2H_2_O as the cation sources yields alloyed (CdS)_x_(CuInS_2_)_1−x_ nanocrystals with a WZ structure. Alternatively, Cu(S_2_CNEt_2_)_2_, In(S_2_CNEt_2_)_3_, and Cd(S_2_CNEt_2_)_2_ are employed as cation sources to produce ZB structure alloyed (CdS)_x_(CuInS_2_)_1−x_ nanocrystals. The band structures of the synthesized alloyed nanocrystals were meticulously elucidated through a synergistic approach employing UV–vis–NIR absorption spectroscopy and Mott–Schottky analysis. The band gaps of WZ alloyed nanocrystals can be systematically tuned across a range of 0.89 eV, and those of the ZB alloyed nanocrystals can also be adjusted within a range of 0.34 eV. These binary–ternary alloyed sulfides demonstrate considerable potential for applications in photocatalysis and photoemission due to the tunable band gap, which can be precisely regulated by controlling the compositional stoichiometry within each crystal.

## Data Availability

The original contributions presented in this study are included in the article/Supplementary Material. Further inquiries can be directed to the corresponding author.

## Supplementary Materials

The following supporting information can be downloaded at https://www.mdpi.com/article/10.3390/nano15211661/s1. Figure S1: Scheme of alloyed (CdS)_x_(CuInS_2_)_1−x_ synthesized; Figure S2: TEM images of WZ alloyed (CdS)_x_(CuInS_2_)_1−x_ nanocrystals; Figure S3: Size distribution of WZ1–5; Figure S4: XRD patterns of WZ1 nanocrystals synthesized with Cd(acac)_2_ and CdCl_2_; Figure S5: TEM images of WZ1 nanocrystals synthesized with Cd(acac)_2_ and CdCl_2_; Figure S6: TEM images of ZB alloyed (CdS)_x_(CuInS_2_)_1−x_ nanocrystals; Figure S7: Size distribution of ZB1–5; Figure S8: TEM images of ZB4 nanocrystals synthesized different reaction conditions; Figure S9: XRD patterns of ZB4 nanocrystals synthesized different reaction conditions; Figure S10: The survey XPS spectra and the XPS spectra of C 1s; Figure S11: Photograph of the WZ and ZB alloyed nanocrystals; Figure S12: M-S plots of WZ alloyed (CdS)_x_(CuInS_2_)_1−x_ nanocrystals; Figure S13: M-S plots of ZB alloyed (CdS)_x_(CuInS_2_)_1−x_ nanocrystals; Table S1: Amounts of CuCl, Cd(AC)_2_·2H_2_O, InCl_3_ used for synthesizing the WZ alloyed (CdS)_x_(CuInS_2_)_1−x_ nanocrystals; Table S2: Amounts of Cu(S_2_CNEt_2_)_2_, In(S_2_CNEt_2_)_3_ and Cd(S_2_CNEt_2_)_2_ used for synthesizing the ZB alloyed (CdS)_x_(CuInS_2_)_1−x_ nanocrystals; Table S3: The atomic ratio and composition x of the as-synthesized WZ alloyed (CdS)_x_(CuInS_2_)_1−x_ nanocrystals were determined by ICP-OES; Table S4: The atomic ratio and composition x of the as-synthesized ZB alloyed (CdS)_x_(CuInS_2_)_1−x_ nanocrystals were determined by ICP-OES.

## Abbreviations

The following abbreviations are used in this manuscript:

CP	Chalcopyrite
WZ	Wurtzite
ZB	Zinc-blende
1-DDT	1-dodecanethiol
OLA	Oleylamine
ODE	1-Octadecene
VB	Valence band
CB	Conduction band
AC	Alternating-current
M-S	Mott−Schottky
E_fb_	Flat-band potentials
FFT	Fast Fourier transform
