# Correction: Zu et al. Phase-Controlled Synthesis of Alloyed (CdS)_x_(CuInS_2_)_1−x_ Nanocrystals with Tunable Band Gap. *Nanomaterials* 2025, *15*, 1661

**DOI:** 10.3390/nano16150966

**Published:** 2026-08-06

**Authors:** Bingqian Zu, Song Chen, Liping Bao, Yingjie Liu, Liang Wu

**Affiliations:** Key Laboratory of Functional Molecular Solids, Ministry of Education, School of Chemistry and Materials Science, Anhui Normal University, Wuhu 241000, China; zubingqian@ahnu.edu.cn (B.Z.); chensong@ahnu.edu.cn (S.C.); 2421011424@ahnu.edu.cn (L.B.); liuyingjie@ahnu.edu.cn (Y.L.)

In the original publication [1], the valance band position (E_VB_) and conduction band position (E_CB_) were miscalculated. Based on the equations E_VB_ = E_f_ − 0.1 V and E_CB_ = E_VB_ + E_g_, the corrected E_VB_ and E_CB_ values are presented in Table 1. Using the corrected E_VB_ and E_VB_ values, Figure 6e,f were corrected.

**Figure 6:**
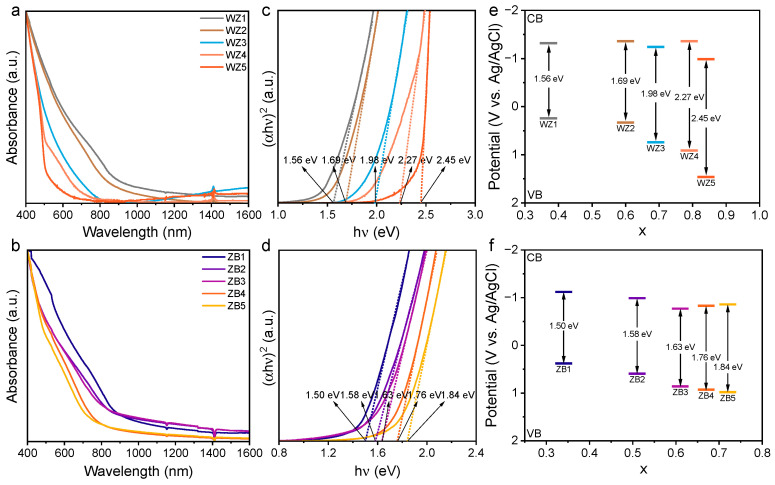
(**a**,**b**) UV-vis-NIR absorption spectra of WZ and ZB alloyed (CdS)_x_(CuInS_2_)_1−x_ nanocrystals with varying compositions, respectively. (**c**,**d**) Plots of linear extrapolation of (*αhν*)^2^ versus photon energy (*hν*) of WZ and ZB alloyed (CdS)_x_(CuInS_2_)_1−x_ nanocrystals with varying compositions, respectively. (**e**,**f**) The schematic diagrams of the electronic band positions of WZ and ZB alloyed (CdS)_x_(CuInS_2_)_1−x_ nanocrystals with varying compositions, respectively.

The authors state that the scientific conclusions are unaffected. This correction was approved by the Academic Editor. The original publication has also been updated.

## Figures and Tables

**Table 1:** The corrected E_VB_ and E_CB_ values.

WZ (CdS)_x_(CuInS_2_)_1−x_					ZB (CdS)_x_(CuInS_2_)_1−x_				
x	E_f_/eV	E_g_/eV	E_VB_/eV	E_CB_/eV	x	E_f_/eV	E_g_/eV	E_VB_/eV	E_CB_/eV
0.37	0.14	1.56	0.24	−1.32	0.34	0.28	1.50	0.38	−1.12
0.6	0.23	1.69	0.33	−1.36	0.51	0.49	1.58	0.59	−0.99
0.69	0.64	1.98	0.74	−1.24	0.61	0.76	1.63	0.86	−0.77
0.79	0.81	2.27	0.91	−1.36	0.67	0.83	1.76	0.93	−0.83
0.84	1.36	2.45	1.46	−0.99	0.72	0.88	1.84	0.98	−0.86
