# Supplementary material for: Phase-Controlled Synthesis of Alloyed (CdS)x(CuInS2)1−x Nanocrystals with Tunable Band Gap
Source: Nanomaterials (Basel). 2025 Nov 1;15(21):1661. doi: 10.3390/nano15211661 (PMC12608115; doi:10.3390/nano15211661)
Supplement: Supplementary file 1 [file nanomaterials-15-01661-s001.zip › nanomaterials-3836736-supplementary.pdf]

Supplementary Materials

Phase-controlled synthesis of alloyed $(CdS)_x(CuInS_2)_{1-x}$ nanocrystals with tunable band gap

*Bingqian Zu, Song Chen, Liping Bao, Yingjie Liu, Liang Wu**

Key Laboratory of Functional Molecular Solids, Ministry of Education, School of Chemistry and Materials Science, Anhui Normal University, Wuhu 241000, China;

*Correspondence and requests for materials should be addressed to Liang Wu (wuliang@ahnu.edu.cn).

Supplementary Experimental Section

Preparation of $Cu(S_2CNEt_2)_2$:

NaS_2CNEt_2 (10mmol) and $Cu(NO_3)_2 \cdot 3H_2O$ (5 mmol) were separately dissolved in 100 mL deionized water. Subsequently, $Cu(NO_3)_2 \cdot 3H_2O$ aqueous solution was slowly added dropwise to the NaS_2CNEt_2 solution under vigorous stirring. After stirring for 30 min, the products are collected via centrifugation, washed at least three times with deionized water and ethanol, and then dried at 60 °C.

Preparation of $Cd(S_2CNEt_2)_2$ and $In(S_2CNEt_2)_3$:

The synthesis procedure employed is identical to that used for $Cu(S_2CNEt_2)_2$.

Preparation of Carbon paper electrodes with nanocrystals:

The carbon paper electrodes were prepared by dropping 60 μL aqueous (30mg nanoparticles dissolved in 1 mL of hexane) onto a cleaned carbon paper. The electrodes were then annealed in a vacuum environment at a temperature of 140 °C for 12 h to remove the organic ligands.

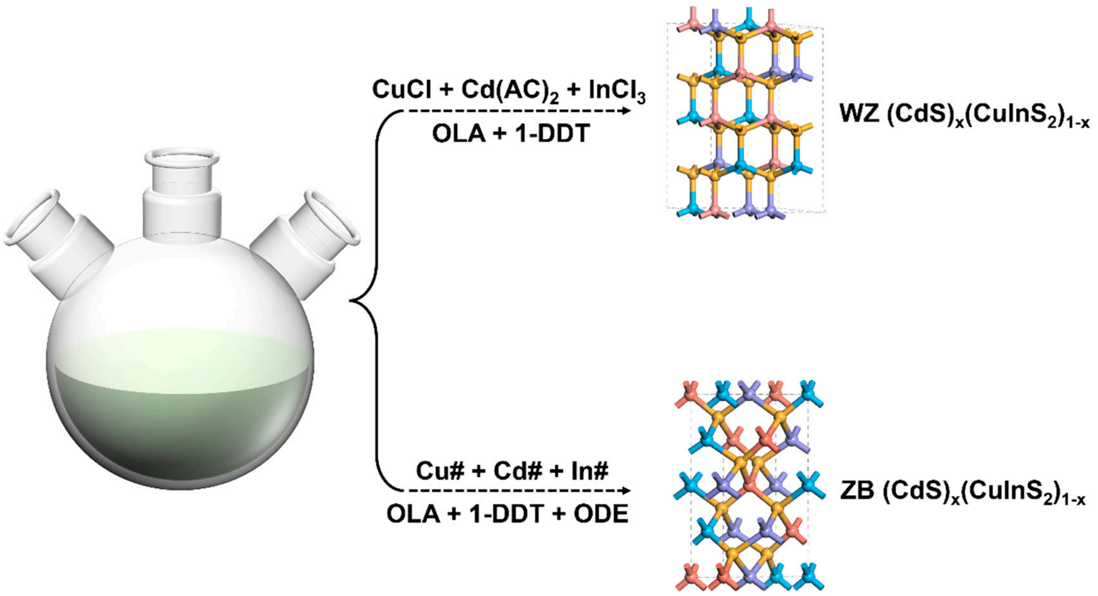

Figure S1. Scheme illustrating the phase- and composition-controlled synthesis of alloyed $(CdS)_x(CuInS_2)_{1-x}$ nanocrystals.

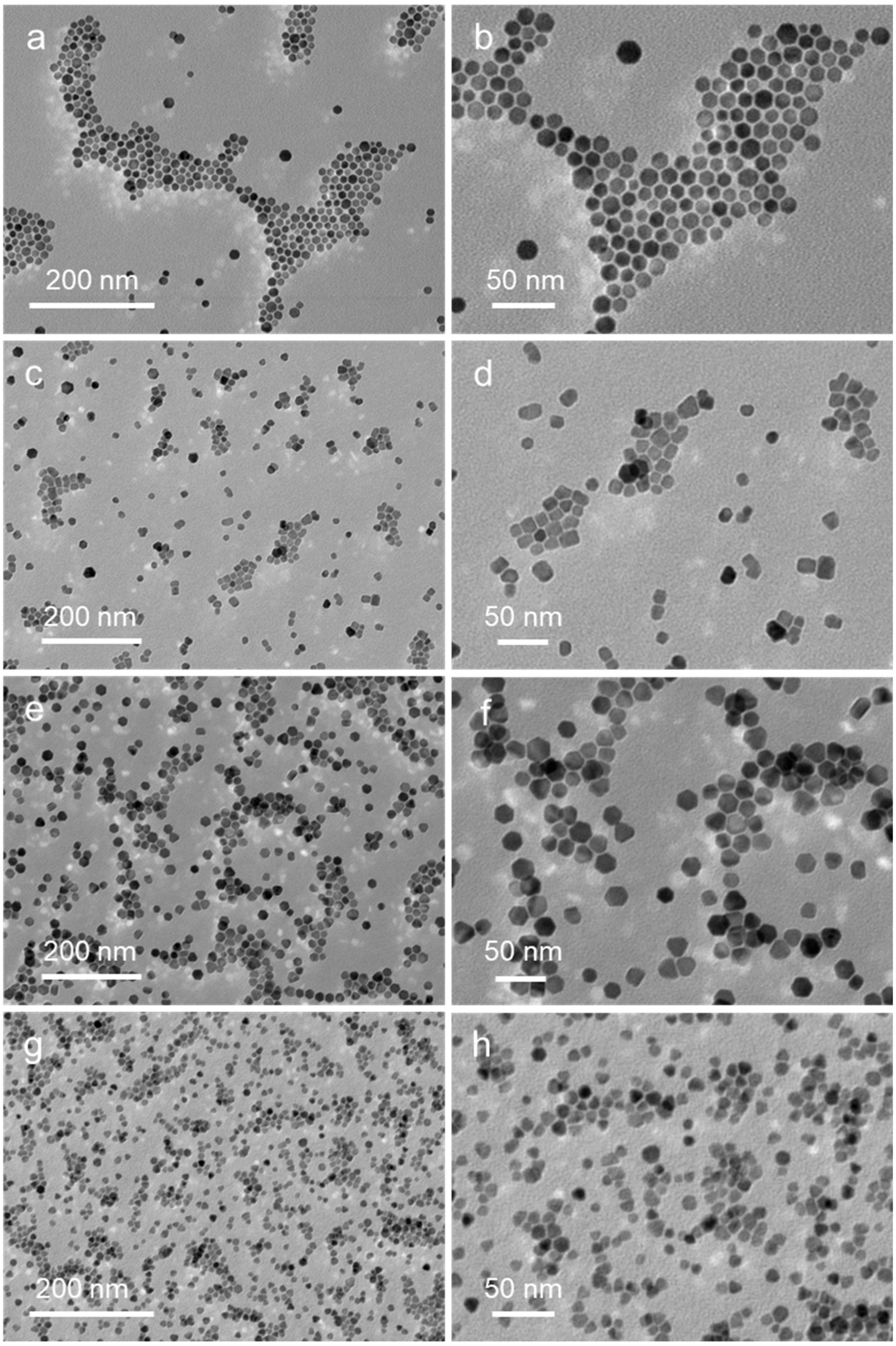

Figure S2. TEM images of the obtained WZ alloyed $(CdS)_x(CuInS_2)_{1-x}$ nanocrystals with various compositions. (a, b) WZ1. (c, d) WZ2. (e, f) WZ4. (g, h) WZ5.

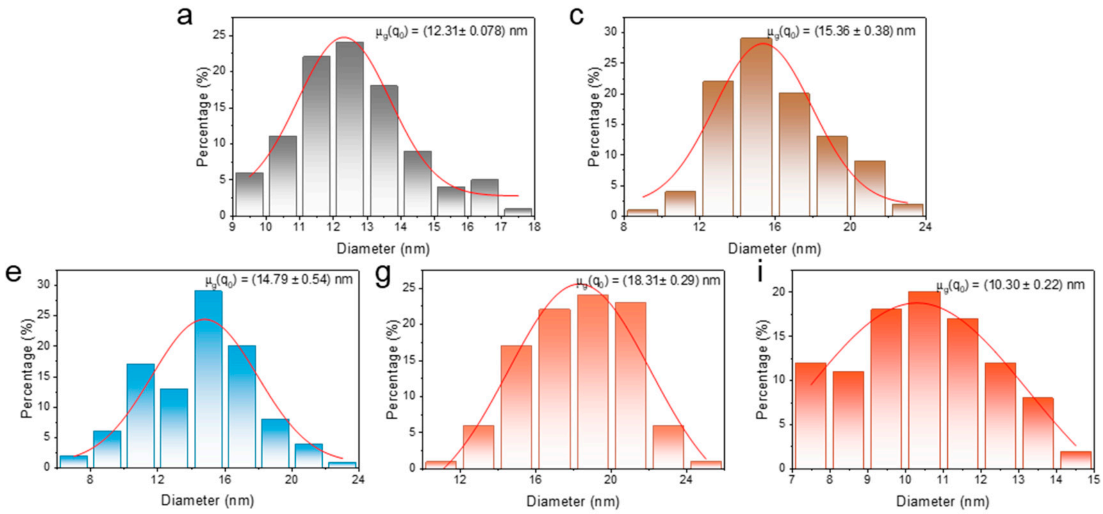

Figure S3. (a-e) Size distribution of the WZ1 to WZ 5 samples, respectively.

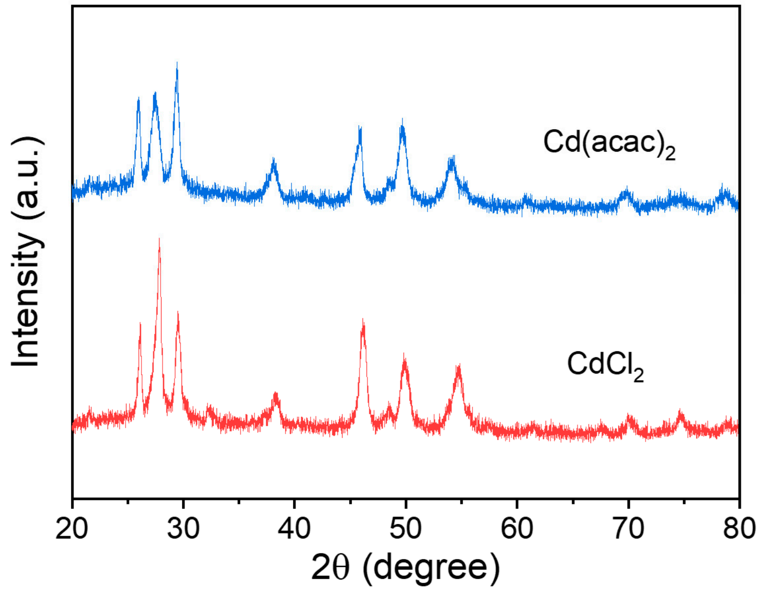

Figure S4. XRD patterns of WZ1 nanocrystals synthesized with $Cd(acac)_2$ and $CdCl_2$, respectively.

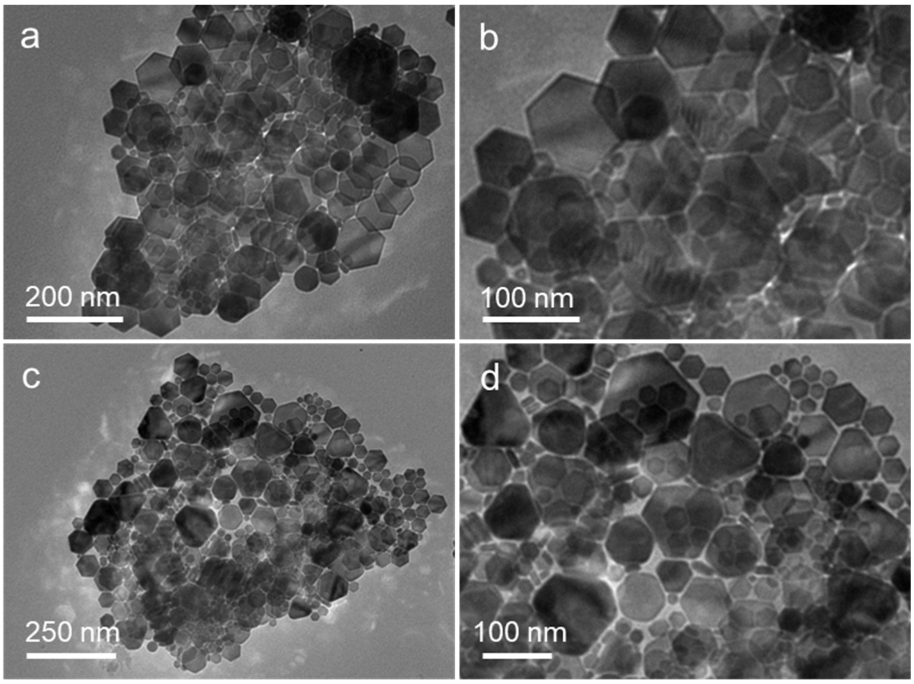

Figure S5. TEM images WZ1 nanocrystals synthesized with different Cd sources. (a, b) $Cd(acac)_2$. (c, d) $CdCl_2$.

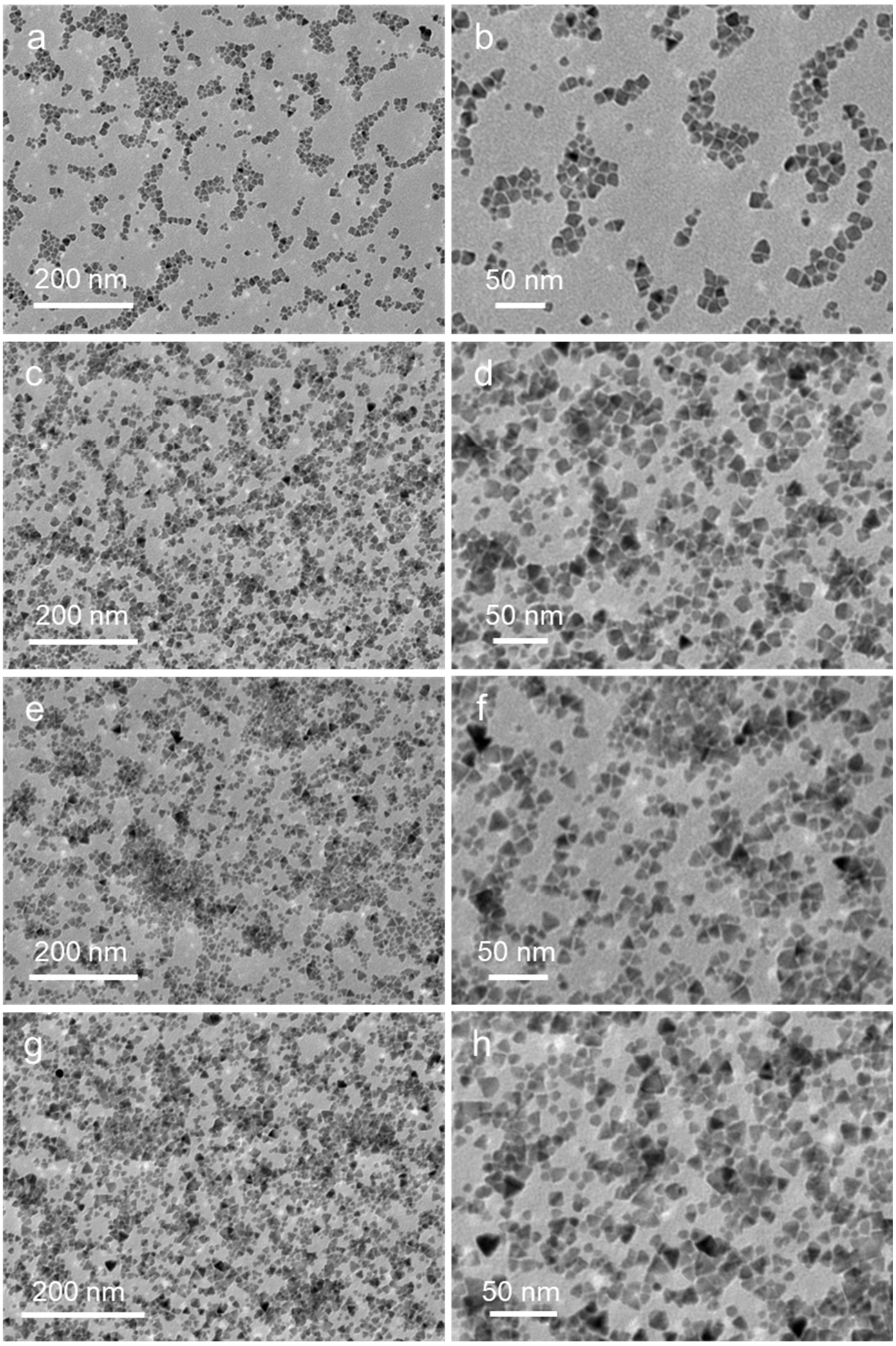

Figure S6. TEM images of the obtained ZB alloyed $(CdS)_x(CuInS_2)_{1-x}$ nanocrystals with various compositions. (a, b) ZB1. (c, d) ZB2. (e, f) ZB4. (g, h) ZB5.

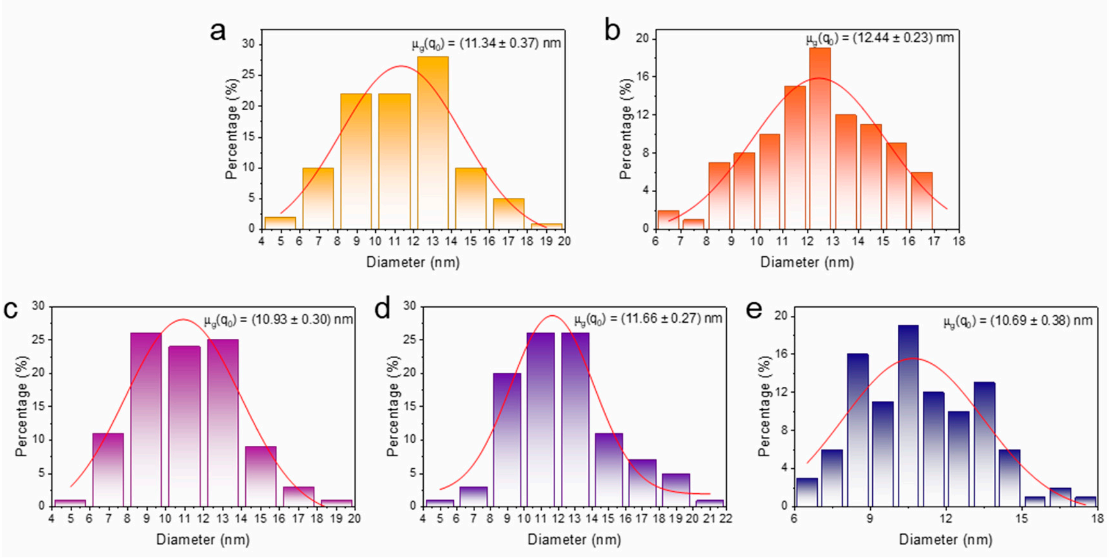

Figure S7. (a-e) Size distribution of the ZB1 to ZB 5 samples, respectively.

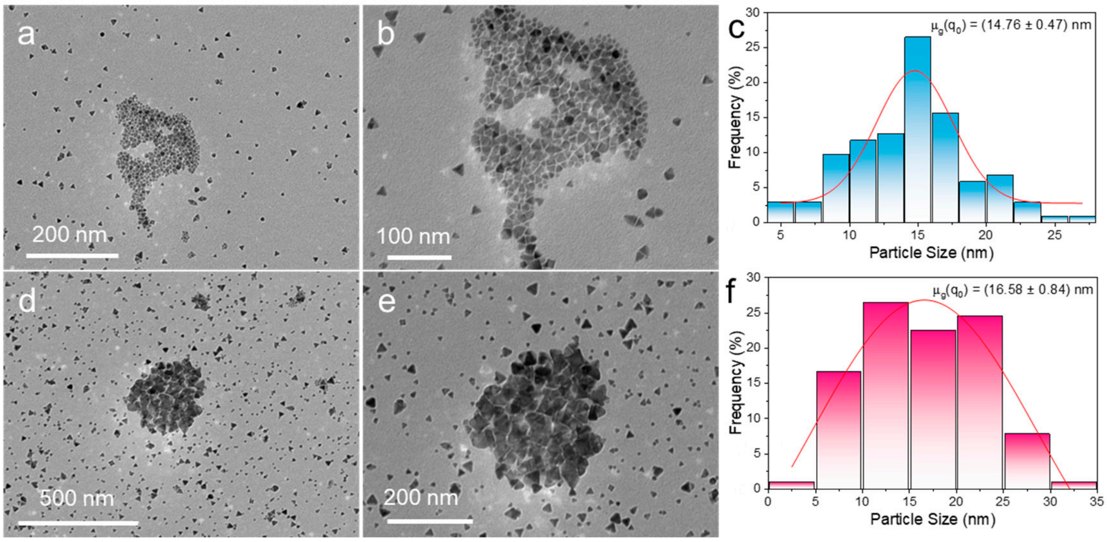

Figure S8. (a-c) TEM images and the size distribution of ZB4 nanocrystals synthesized using 10 mL of OLA and 1 mL 1-DDT at 280 ºC for 60 min. (d-f) TEM images and the size distribution of ZB4 nanocrystals using 0.2 mmol $Cu(S_2CNEt_2)_2$, 0.8 mmol $Cd(S_2CNEt_2)_2$, 0.2 mmol $In(S_2CNEt_2)_3$, 8 mL of OLA, 8 mL of 1-DDT and 4 mL of ODE.

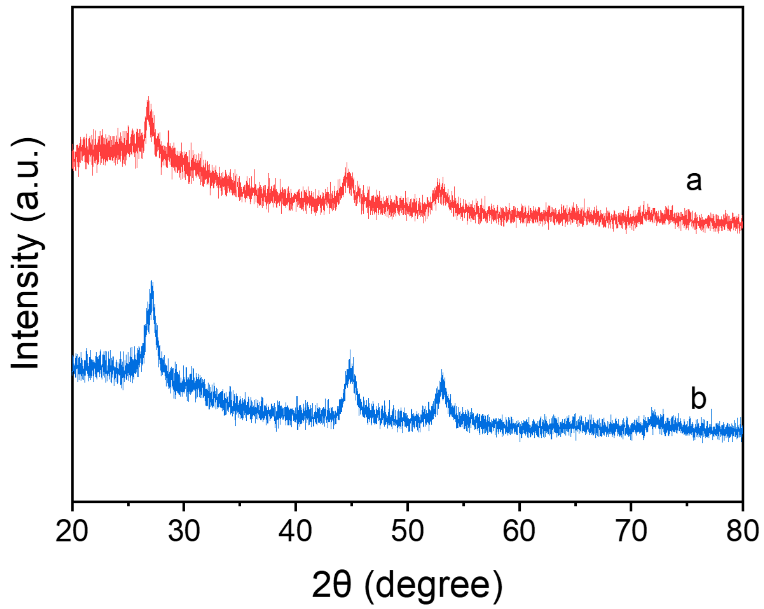

Figure S9. (a) XRD pattern of ZB4 nanocrystals synthesized using 10 mL of OLA and 1 mL 1-DDT at 280 °C for 60 min. (b) XRD pattern of ZB4 nanocrystals using 0.2 mmol $Cu(S_2CNEt_2)_2$, 0.8 mmol $Cd(S_2CNEt_2)_2$, 0.2 mmol $In(S_2CNEt_2)_3$, 8 mL of OLA, 8 mL of 1-DDT and 4 mL of ODE.

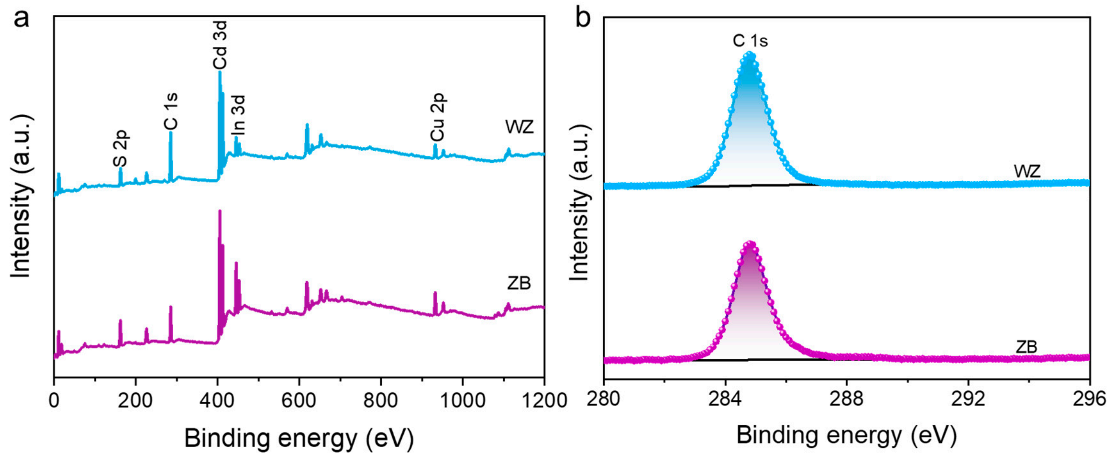

Figure S10. (a) The survey XPS spectra of WZ3 and ZB3. (b) The XPS spectra of C 1s.

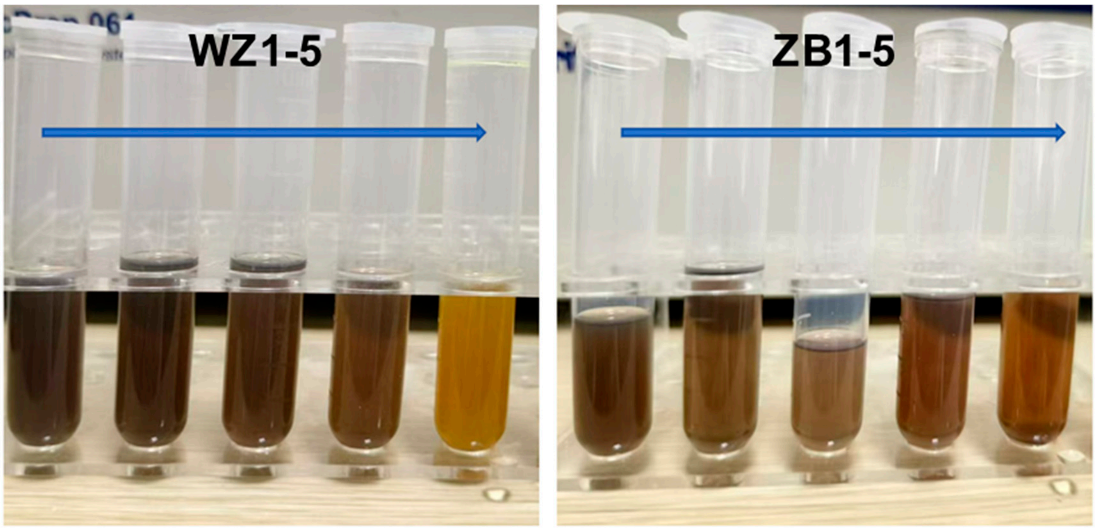

Figure S11. Photograph of the WZ alloyed $(CdS)_x(CuInS_2)_{1-x}$ and ZB alloyed $(CdS)_x(CuInS_2)_{1-x}$ nanocrystals dispersed in hexane, respectively.

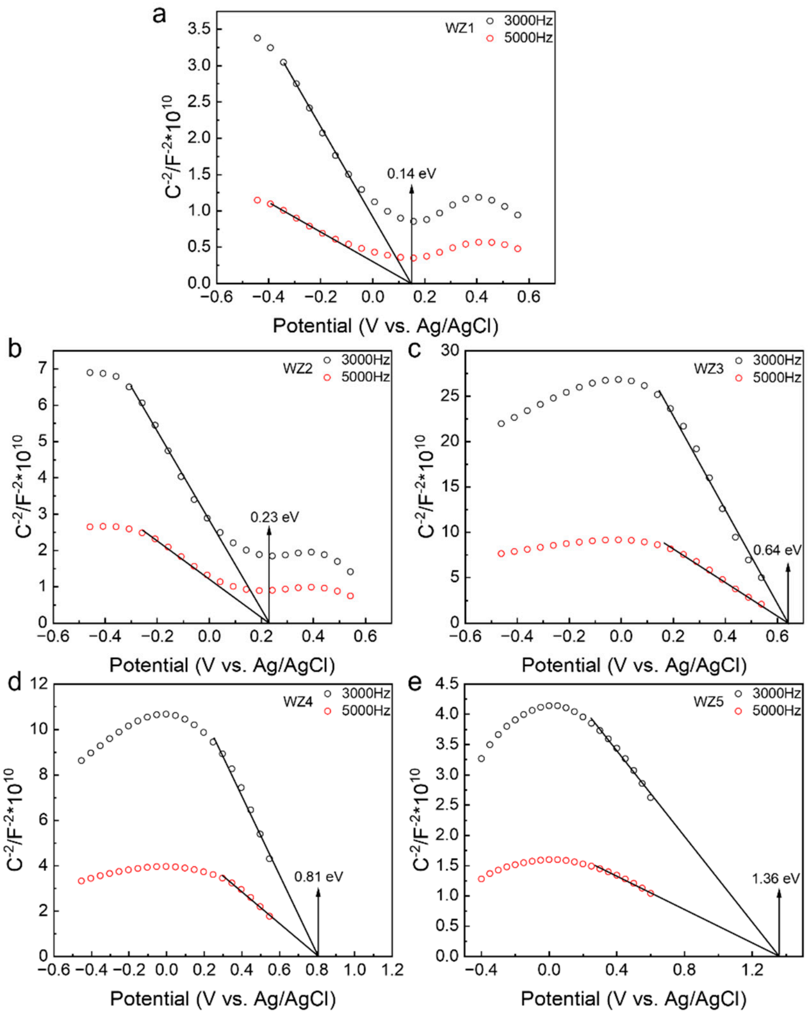

Figure S12. Representative M-S plots of WZ alloyed $(CdS)_x(CuInS_2)_{1-x}$ nanocrystals with various compositions. (a) WZ1. (b) WZ2. (c) WZ3. (d) WZ4. (e) WZ5.

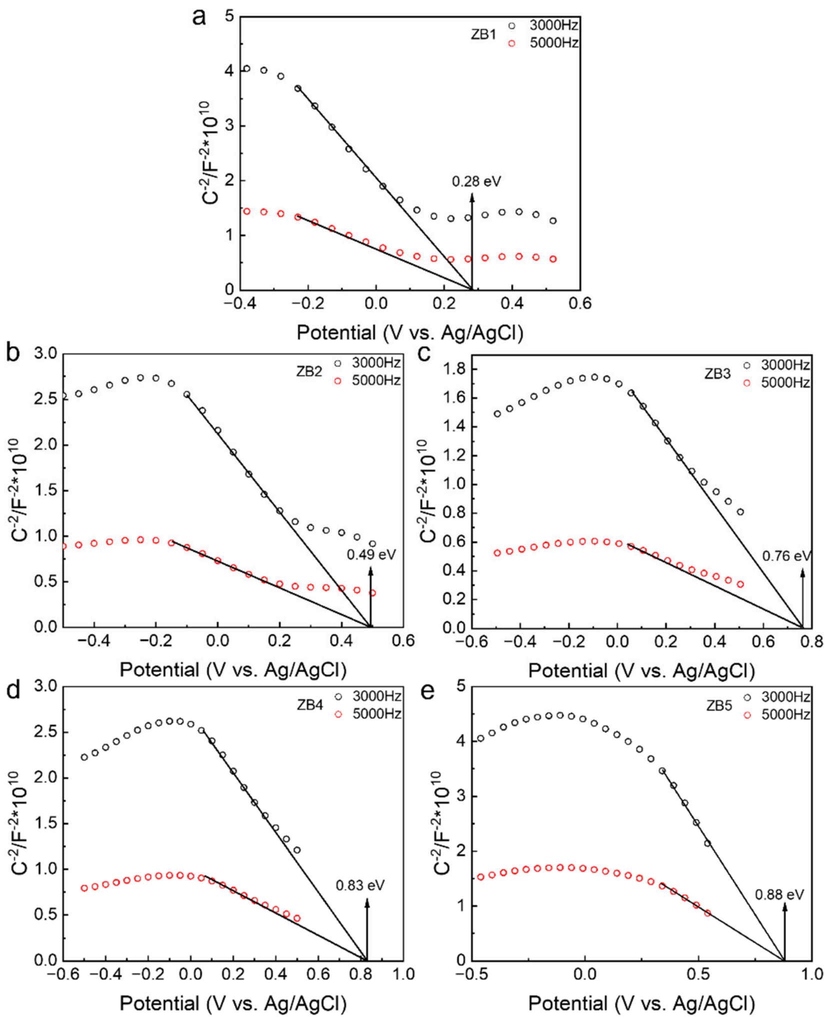

Figure S13. Representative M-S plots of ZB alloyed $(CdS)_x(CuInS_2)_{1-x}$ nanocrystals with various compositions. (a) ZB1. (b) ZB2. (c) ZB3. (d) ZB4. (e) ZB5.

Table S1. Amounts of CuCl, $Cd(AC)_2 \cdot 2H_2O$, $InCl_3$ used for synthesizing the WZ alloyed $(CdS)_x(CuInS_2)_{1-x}$ nanocrystals with various compositions.

Sample	CuCl (mmol)	$Cd(AC)_2 \cdot 2H_2O$ (mmol)	$InCl_3$ (mmol)
**WZ1**	0.5	0.5	0.5
**WZ2**	0.5	1.0	0.5
**WZ3**	0.5	1.5	0.5
**WZ4**	0.5	2.0	0.5
**WZ5**	0.5	2.5	0.5

Table S2. Amounts of $Cu(S_2CNEt_2)_2$, $Cd(S_2CNEt_2)_2$, and $In(S_2CNEt_2)_3$ used for synthesizing the ZB alloyed $(CdS)_x(CuInS_2)_{1-x}$ nanocrystals with various compositions.

Sample	$Cu(S_2CNEt_2)_2$ (mmol)	$Cd(S_2CNEt_2)_2$ (mmol)	$In(S_2CNEt_2)_3$ (mmol)
**ZB1**	0.1	0.1	0.1
**ZB2**	0.1	0.2	0.1
**ZB3**	0.1	0.3	0.1
**ZB4**	0.1	0.4	0.1
**ZB5**	0.1	0.5	0.1

Table S3. The atomic ratio and composition x of the as-synthesized WZ alloyed $(CdS)_x(CuInS_2)_{1-x}$ nanocrystals were determined by ICP-OES.

Sample	Cu:Cd:In	x
**WZ1**	1 : 0.58 : 0.62	0.37
**WZ2**	1 : 1.52 : 0.59	0.60
**WZ3**	1 : 2.21 : 0.37	0.69
**WZ4**	1 : 3.81 : 0.31	0.79
**WZ5**	1 : 5.29 : 0.49	0.84

Table S4. The atomic ratio and composition x of the as-synthesized ZB alloyed $(CdS)_x(CuInS_2)_{1-x}$ nanocrystals were determined by ICP-OES.

Sample	Cu:Cd:In	x
**ZB1**	1 : 1.02 : 1.00	0.34
**ZB2**	1 : 2.10 : 1.09	0.51
**ZB3**	1 : 3.15 : 1.10	0.61
**ZB4**	1 : 4.11 : 1.02	0.67
**ZB5**	1 : 5.12 : 1.03	0.72
